# Understanding the Fresh Produce Safety Challenges

**DOI:** 10.3390/foods6030023

**Published:** 2017-03-21

**Authors:** Malik Altaf Hussain, Ravi Gooneratne

**Affiliations:** Department of Wine, Food and Molecular Biosciences, Lincoln University, Lincoln 7647, New Zealand; ravi.gooneratne@lincoln.ac.nz

Consumption of fresh fruits and vegetables is important for a balanced diet and healthy life-style. However, contamination of fresh produce is emerging as a major food safety challenge. In recent years, contaminated produce has been implicated in many foodborne outbreaks throughout the world. These foodborne outbreaks are not only a burden on public health but also cause heavy economic loss to the food industry [1]. A recent report by Center for Science in the Public Interest (CSPI) showed that the highest number of outbreaks was attributed to fresh produce commodity in the USA during 2002–2011 [2]. It is estimated that fresh produce causes the greatest number of illnesses and the largest average number of illnesses per outbreak. 

With the push to increase consumption of fresh produce for healthy living, there is pressure on producers to focus even more on hygiene to minimize exposure to food hazards. A risk exists especially if the fresh produce is grown outdoors in the field. Fresh produce can be contaminated by physical, chemical, and biological hazards. Physical hazards could include dust, sand, wood and metal pieces. Chemical hazards include chemicals in packaging and/or pesticides used on the farm. Biological hazards include microbiological contaminants such as *Escherichia coli*, *Salmonella*, *Listeria monocytogenes* and other pathogenic microorganisms in the soil. The greatest risk is when vegetables and fruits are consumed without being washed. This is an important consideration to bear in mind by the growers of fresh produce. The worst-case scenario is when the biological/chemical contamination is not washed off the produce by both the farmer and the consumer. In many countries, there are regulations and schemes to train and educate producers. Therefore, nowadays, most farmers wash their produce and many large growers even go further to reduce hazards by implementing a complete food safety management system.

Several groups of microorganisms can colonize or contaminate fruits and vegetables at any point throughout the food supply chain. Pathogenic microorganisms such as *E. coli* O157:H7, *Salmonella*, *L. monocytogenes* and norovirus are commonly associated with contaminated fresh produce. Various types of fresh produce including cantaloupe, strawberries, mangos, leafy green vegetables, lettuce, salad mixes, sprouts, cabbage, cut celery and radishes are potential vehicles for transmission of these human pathogens. Globally, many fresh produce linked outbreaks occurred over the last few years including an outbreak of *E. coli* O157:H7 after eating contaminated packaged baby spinach in EU countries (2006); an *E. coli* outbreak due to contaminated cucumber in Germany and other EU countries (2011); an outbreak of *Cryptosporidium* infection traced to bagged salads in the UK (2012); an outbreak of *L. monocytogenes* due to contaminated prepacked salad products in the USA (2016); and a *Salmonella* outbreak linked to lettuce in pre-packaged salads in Australia (2016). 

Bacterial contamination of fresh produce such as lettuce, cabbage, other leafy vegetables, root vegetables, asparagus, broccoli, and cauliflower is well known and some of it could be attributed to improper handling practices and poor storage conditions. Innovative business opportunities and product diversity that appeal to the consumer may also increase food safety risks. Cut fruits and vegetables have a higher microbial risk profile than the ‘whole’ produce. Therefore, it is not surprising that delicatessen salads made up of the same vegetables are more contaminated. The presence of excessive levels of bacteria suggest poor hygiene conditions. One of the problems associated with fresh produce is that, unlike tinned or packaged foods, there is a lack of information on the shelf-life and expiration dates. 

There are risks associated with fresh produce sold by street vendors. For example, potatoes exposed directly to the sun can result in solanine production and consumption of foods containing solanine can result in nausea, vomiting, diarrhea, headache, dizziness, fever and in more severe cases, hallucinations, paralysis and even death. When exposed to rain and sun at 20 °C, *Salmonella, E. coli* O157-H7 or *L. monocytogenes* will multiply to toxic levels in cauliflower. A comprehensive study by Farber and Peterki [3] showed that *L. monocytogenes* can survive in lettuce juice even at 4 °C. This pathogen has been identified in many ingredients of the green salads and also in pre-packed salads. *L. monocytogenes* can even grow at 12 °C on fresh blueberries stored under a controlled atmosphere [4]. Bacterial cells appear to increase by about 2–4-fold in 6 days on vegetables such as the asparagus, broccoli, and cauliflower at refrigerator temperature. However, at a refrigerated temperature of 4 °C, they do not grow well and in fact decrease in vegetables such as broccoli and cauliflower by about 0.5-fold after 14 days. Hence the most important message is to store vegetables under a controlled low temperature that not only increases shelf-life but also reduces bacterial growth considerably.

Another challenge to food scientists is the emergence of antimicrobial resistant (AMR) bacterial strains in foods [5] including fresh produce. This issue has emerged as an important and growing public health concern and an economic problem in many countries over the last two decades. So, it is necessary to understand the pathways of antimicrobial resistant pathogens contamination and act to minimize their introduction and occurrence in fresh produce. To address the AMR issue effectively, measures such as developing public, industry and government cooperation, introducing various surveillance programs, and strengthening the food safety systems are essential.

In conclusion, it is important to understand the nature of fresh produce safety challenges, contamination sources, risks to the consumer, and approaches to eliminate or reduce the level of contaminants. Scientific understanding is rapidly evolving in this important area of food safety. Two recently published articles [6,7] provide an insight into the scientific and technical importance of fresh produce safety. This Special Issue of *Foods* on ‘fresh produce safety’ invites manuscripts on all aspects of safe supply and consumption of fresh fruits and vegetables.
